# Imaging of Ocular Angle Structures with Fourier Domain Optical Coherence Tomography

**DOI:** 10.5005/jp-journals-10008-1141

**Published:** 2013-05-09

**Authors:** Sanjay Asrani, Mei Young, Jing Xu, Marinko V Sarunic

**Affiliations:** Professor, Department of Ophthalmology, Duke Eye Center, Duke University, Durham, North Carolina, USA; Biomedical Engineer, Department of Ophthalmology and Visual Sciences, University of British Columbia, British Columbia, Canada; PhD Candidate, School of Engineering Science, Simon Fraser University British Columbia, Canada; Associate Professor, School of Engineering Science, Simon Fraser University British Columbia, Canada

**Keywords:** Optical coherence tomography, Anterior eye segment, Trabecular meshwork.

## Abstract

**Background:** Imaging of the Schlemm's Canal is complicated by the small physiological size and the location several hundred microns beneath the sclera. Noninvasive imaging of Schlemm's canal and Trabecular Meshwork (TM) *in vivo* with Fourier Domain Optical Coherence Tomography (FD OCT) can provide clinicians with a powerful tool to visualize ocular angular structures crucial for glaucoma management.

**Purpose:** To investigate the appearance of Schlemm's canal and TM on FD OCT images.

**Methods:** FD OCT images of the Schlemm's canal and TM were obtained with three different wavelengths using prototype FD OCT systems in a normal volunteer. FD OCT images using the 1310 nm wavelength prototype were obtained in three representative cases of glaucoma surgery performed on angle structures.

**Results:** The longer imaging depth and deeper tissue penetration of the 1310 nm system provided the clearest image of the TM and Schlemm's canal in the normal patient. In case 1, images pre- and post-trabectome surgery clearly showed the location and appearance of TM. In case 2, images post-canaloplasty surgery showed the location and appearance of Schlemm's canal. In case 3, images pre- and post-trabeculotomy surgery further confirms the appearance and location of the Schlemm's canal and TM.

**Conclusion:** Operating wavelength of the FD OCT system and exact location of the scan across different meridians minimally affects the appearance of the ocular anatomy. The postoperative images of three angle glaucoma surgeries confirmed the location of Schlemm's canal and TM.

**How to cite this article:** Asrani S, Young M, Xu J, Sarunic MV. Imaging of Ocular Angle Structures with Fourier Domain Optical Coherence Tomography. J Current Glau Prac 2013;7(2):85-87.

## INTRODUCTION

Noninvasive visualization of the Schlemm's canal and trabecular meshwork (TM) *in vivo* would permit improved research and assist in the clinical management of glaucoma. Imaging of the SC in particular is complicated by the small physiological size and the location several hundred microns beneath the sclera. The ability to image SC has been demonstrated using high frequency ultrasound (UBM 50 MHz),^1^ time domain optical coherence tomography (OCT)^2^ and more recently, swept source fourier domain (FD) OCT systems. The latter have provided higher speed and higher resolution images of the ocular angle permitting clearer visualization of the SC and the TM.^3,4^ Commercial FD OCT systems have also depicted SC (SC) and TM differently and this may lead to some confusion about the structures identified.

In this report, we depict SC and TM with three different wavelengths using prototype FD OCT systems in a normal volunteer to show the variation in the images obtained. We also depict the SC and TM location in a few representative cases of glaucoma surgery performed on angle structures to confirm that the structures labeled in the normal anatomy are indeed the same.

**Table 1:** **Table 1:** OCT properties operating at different wavelengths

		*Operating wavelength*					
*Properties*		*830 nm*		*1,060 nm*		*1,310 nm*	
Axial resolution (tissue)		~9μm		~9μm		~3μm	
Tissue scattering (relative)		High		Medium		Low	
Water absorption (relative)		Low		Medium		High	
Laser source		Super luminescent diode (Superlum, Moscow, Russia)		Wavelength-swept laser (Micron Optics Inc., Atlanta, GA)		Wavelength-swept laser (Micron Optics Inc., Atlanta, GA)	

## MATERIALS AND METHODS

We investigated FD OCT systems for imaging the SC and TM at three wavelengths commonly used in OCT: 1,310 nm swept source, 1,060 nm swept source and 830 nm spectral-domain OCT. The differences of the three systems are described in Table 1.

**Figs 1A to F:**
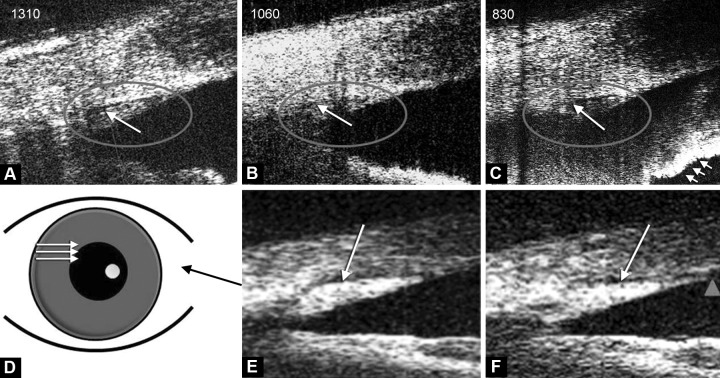
Images of the Schlemm's canal with three different wavelengths (A to C) and with the 1,310 nm wavelength swept OCT system at different points on the subject's eye (D to F). The shapes of the regions identified as the TM and the Schlemm's canal (long arrow) changes at different points in the elevation

B-scan images of the ocular angle from same volunteer with all three systems are shown in Figures 1A to C. The same features are observed with all systems, with minor differences due to the different tissue back scattering properties of imaging wavelength.

Figure 1D is a schematic of the locations of multiple B-scans acquired on a subject's eye. The shape difference of SC at two locations is presented through comparison of Figures 1E and F. Despite being from the same eye and from the same system, the shape of SC appears different in both images. In Figure 1F, the optical reflectivity of the tissue around the TM creates an artifactual appearance of the SC is communicating with the anterior chamber (triangle).

## CASE REPORTS

The 1,310 nm wavelength swept FD OCT system was used to study the angle in 143 patients. The SC and TM are shown in three representative cases where surgical procedures had been performed on the angle structures.

**Figs 2A and B:**
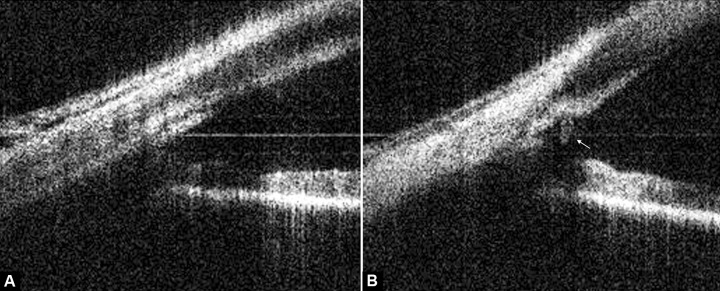
Ocular anterior chamber angle: (A) Pretrabectome surgery and (B) post-trabectome surgery

### Case 1

An elderly male with moderate glaucoma in his right eye underwent trabectome surgery. The image prior to the surgery shows the intact TM in Figure 2A. The image taken 2 days after surgery is presented in Figure 2B and shows the incision into the TM with the SC exposed to the anterior chamber with two cut edges of the meshwork on either side (arrow).

### Case 2

**Fig. 3:**
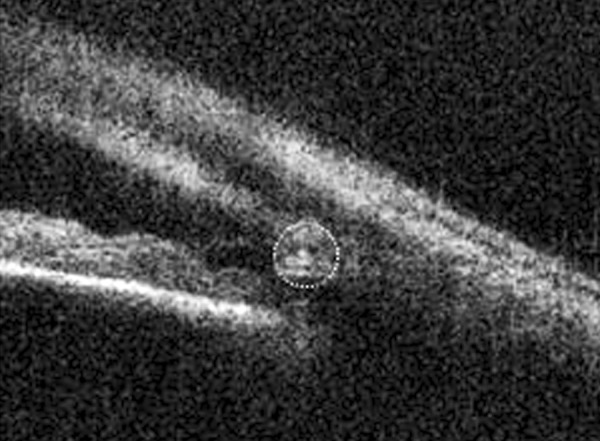
Proline suture observed as a bright round reflection (in the center of the circle) within the Schlemm's canal following canaloplasty surgery

A female in her 70's with moderate glaucoma in her left eye underwent canaloplasty surgery which involves insertion of a 10'0 proline suture for 360° of the canal. Figure 3, taken 3 months after the surgery, shows the cross-section of the proline suture within the canal.

**Figs 4A and B:**
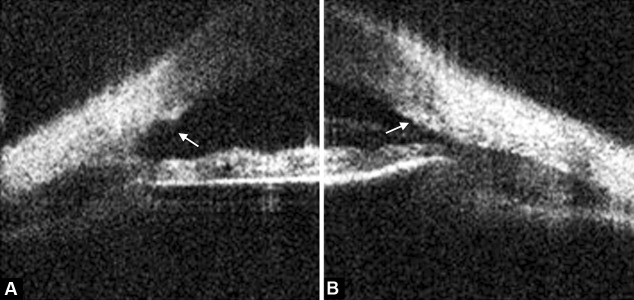
(A) The TM was surgically removed and (B) the TM can be seen in the normal anatomy from the contralateral eye

### Case 3

A young boy who had undergone trabeculotomy in his right eye for congenital glaucoma at age 3. At the expected location of the SC and TM, no remnants of the TM are seen. Instead, a concave dark area is visualized Figure 4A (arrow). This results from the removed TM during the surgery and the exposed SC to the anterior chamber. In the contralateral eye, the intact TM is visualized Figure 4B.

## CONCLUSION

Noninvasive imaging of the ocular angle with FD OCT permits visualization of the SC and TM. Selection of the OCT operating wavelength and exact location of the scan across different meridia minimally affects the appearance of the ocular anatomy. The postoperative images (using 1,310 nm swept source FD OCT) of three angle glaucoma surgeries confirmed the location of SC and TM.
